# Parasites and steroid hormones: corticosteroid and sex steroid synthesis, their role in the parasite physiology and development

**DOI:** 10.3389/fnins.2015.00224

**Published:** 2015-06-30

**Authors:** Marta C. Romano, Pedro Jiménez, Carolina Miranda-Brito, Ricardo A. Valdez

**Affiliations:** ^1^Departamento de Fisiología, Biofísica y Neurociencias, CINVESTAV del IPNMexico city, Mexico; ^2^Centro de Investigación en Reproducción Animal, CINVESTAV-UATTlaxcala, Mexico

**Keywords:** parasites, steroid synthesis, sex steroids, corticosteroids, steroidogenic enzymes, *Taenias*, cysticerci

## Abstract

In many cases parasites display highly complex life cycles that include the penetration and permanence of the larva or adults within host organs, but even in those that only have one host, reciprocal, intricate interactions occur. Evidence indicates that steroid hormones have an influence on the development and course of parasitic infections. The host gender's susceptibility to infection, and the related differences in the immune response are good examples of the host-parasite interplay. However, the capacity of these organisms to synthesize their own steroidogenic hormones still has more questions than answers. It is now well-known that many parasites synthesize ecdysteroids, but limited information is available on sex steroid and corticosteroid synthesis. This review intends to summarize some of the existing information in the field. In most, but not all parasitosis the host's hormonal environment determines the susceptibility, the course, and severity of parasite infections. In most cases the infection disturbs the host environment, and activates immune responses that end up affecting the endocrine system. Furthermore, sex steroids and corticosteroids may also directly modify the parasite reproduction and molting. Available information indicates that parasites synthesize some steroid hormones, such as ecdysteroids and sex steroids, and the presence and activity of related enzymes have been demonstrated. More recently, the synthesis of corticosteroid-like compounds has been shown in *Taenia solium* cysticerci and tapeworms, and in *Taenia crassiceps* WFU cysticerci. In-depth knowledge of the parasite's endocrine properties will contribute to understand their reproduction and reciprocal interactions with the host, and may also help designing tools to combat the infection in some clinical situations.

## Parasites and host interplay: steroid hormones influence parasite development and survival

Progesterone, androgens, and estrogens are present and have critical roles in the vertebrate reproduction and metabolism, but the influence and occurrence of steroid hormones in invertebrates had received less attention (Lafont, 2000) the interplay between the parasite and the host defines the intensity of parasite infections. In many cases, the presence of parasites in the host changes its endocrine equilibrium due to the activation of the immune system response, which finally affects the endocrine system through the influence of cytokines and growth factors released by the immune cells. It is now widely accepted that corticosteroids and sex-related hormones influence the immune response (Roberts et al., 2001; Coutinho and Chapman, 2011; Reyes-Hernandez et al., 2013), thereafter any endocrine perturbation initiated by an infection will change the neuroendocrine equilibrium. These hormonal changes resulting from a spontaneous or experimental infection, affect the parasitic charge, the course of the infection and the parasite's survival (Barthelemy et al., 2004). On the other hand, some parasite infections disrupt the host endocrine system, in a noteworthy example, the dromedary bull, *Camelus dromedarius*, parasitized with *Tripanosoma evansi*, presents changes in the sexual steroid plasmatic concentrations, as well as in the semen characteristics (Al Qarawi, 2005). Furthermore, the male Fence Lizard (*Sceloporus occidentalis*) infected with the malarial parasite *Plasmodium mexicanum* shows several reproductive pathologies, such as fewer courtship and decreased testosterone levels (Dunlap and Schall, 1995) and the *Toxoplasma gondee* infection enhances testicular steroidogenesis in rats (Lim et al., 2013). Interestingly, it has been shown that the host hormonal environment determines the susceptibility, the course, and severity of many parasite infections, and therefore a clear dichotomy in infection susceptibility between males and females had been observed (Morales-Montor et al., 2004). A rich estrogen environment facilitates *Taenia crassiceps* cysticerci proliferation, blocking thus the P450-aromatase with fadrozole decreased parasite load (Morales-Montor et al., 2002). Parasites may also alter the host's reproductive behavior (Thompson and Kavaliers, 1994) as had been also shown in *Taenia crassiceps* ORF infected male mice (Morales et al., 1996).

Steroids and steroid synthesis inhibitors influence the fertility of *Schistosoma mansoni in vitro* (Morrison et al., 1986) while progesterone, makisterone, and ecdysone increased the length of the larvae *Ascaris suum*, a roundworm swine parasite (Fleming, 1985), while progesterone, 17β-estradiol, and testosterone added to the culture medium enhanced the number of *Plasmodium falciparum* gametocytes (Lingnau et al., 1993). Progesterone, but not testosterone decreased the *in vitro* molting process of *Trichinella spiralis* (Hernández-Bello et al., 2011) and *T. crassiceps* ORF cysticerci cell proliferation was increased by physiological concentrations of testosterone, and 17β-estradiol added to the culture media (Romano et al., 2003), while high concentrations inhibited its reproduction (Escobedo et al., 2004).

## The role of corticosteroids in the host-parasite interplay

It is well-known that non-physiological stress situations, such as social isolation, infections, persecution, etc., increase serum corticosteroids levels with the consequent impairment of the immune response. The interplay host-parasite is not the exception, for example social stress caused by female isolation increased blood *Typanosoma cruzi* infection in the wild mouse *Calomys callosus*, which showed body weight loss and impaired immune response (Santos et al., 2008), whereas the hypothalamus-pituitary-interregnal axis of rainbow trout was altered by the parasite haemoflagelate *Cryptobia* infection (Madison et al., 2013). In addition, the infection with *Anguillicola novaezelandiae* affects cortisol levels in the European Eel (Dangel et al., 2014). Frequently, the host and the parasites are affected in the course of infection, as in Bluegill Sunfish (*Lepomis macrochirus*) infected with *Utterbackia imbecillis*, which results in plasma cortisol increment in the host, favoring thus the parasite juvenile metamorphosis (Dubansky et al., 2011). On the other hand, *in vivo* treatment of rats with cortisol increased the growth rate of the protozoan parasite *Toxoplasma gondii* in isolated peritoneal macrophages (Wang et al., 2014).

## Direct effects of glucocorticoids on parasite growth

Aside from the influence of corticosteroids in the course of parasitic infections, it had been shown that these hormones directly influence parasite's growth. For instance, cortisol and dexamethasone increase the *in vitro* multiplication of the haemoflagellate *Cryptobia salmonistica*, possible by their interaction with glucocorticoid receptor-like protein (Li et al., 2013, 2014). In this regard, dehydroepiandrosterone (DHEA) addition to the culture media decreased, while cortisol increased the *in vitro* growth and viability of *Entamoeba histolytica* (Carrero et al., 2006). We had shown that corticosterone and dexamethasone increase the capacity of *T. crassiceps* WFU cysticerci to synthesize androgens and estrogens, hormones that favor the parasite reproduction (Hinojosa et al., 2011).

## Parasites synthesize steroid hormones

### Lipids and steroid hormones

Lipids, and particularly cholesterol and their metabolites, are required and synthesized by some parasites. Bansal et al. (2005) reviewed the requirement for lipids, particularly cholesterol, by pathogens like protozoa (Leishmaniosis, Malaria, and Toxoplasmosis). It has recently been stated that cholesterol exerts many of its functions by maintaining a specialized type of membrane domain called “lipid rafts” in a functional state. These domains are rich in cholesterol and sphingolipids and could be involved in signal transduction and in the entry of pathogens to the host cells (Simons and Toomre, 2000).

The incorporation and utilization of arachidonic acid, linoleic acid, 3-sn-phosphatidycholine, tripalmitylglycerol, and cholesterol by adult *Schistosoma mansoni* was demonstrated by Rumjanek and Simpson (1980). These parasites exchange cholesterol and other metabolites during reproduction (Popiel and Basch, 1986; Silveria et al., 1986), while cholesterol is absorbed by the hydatid cysts of *Echinococcus granulosus* (Bahr et al., 1979). On the other hand, parasites like *Trypanosome cruzi* and some species of *Leishmania* cannot use cholesterol but they synthesize ergosterol and related 24-alkylated sterols. Furthermore, *Leishmania* has a strict requirement for ergosterol for their survival and growth (Urbina et al., 2002; Magaraci et al., 2003; Bazin et al., 2006).

### Ecdysteroids

Several parasites synthesize ecdysteroids, steroid hormones that are essential for arthropod molting; the capacity to synthesize these hormones has been used to classify cestodes (de Loof and Huybrechts, 1998). However, the role of these steroids in parasites remains obscure. Ecdysterone production has been reported in a variety of helminth species, among them, the nematodes *Dirofilaria immitis*, *Brugia pahangi*, *Ascaris suum*, and *Anisakis simplex*, the cestodes: *Moniezia expansa*, *Echinococcus granulosus*, and *Hymenolepis diminuta* and the trematodes: *Fasciola hepatica* and *Schistosoma mansoni* (Mendis et al., 1983, 1984; Fleming, 1985; Cleator et al., 1987; Evershed et al., 1987; Mercer et al., 1987a,b, 1990; Foster et al., 1992). In male and female, *Dirofilaria immitis*, and in female, *Ascaris suum* ecdysteroids were concentrated in the reproductive system, whereas the presence of ecdysteroids in the eggs of *Schistosoma mansoni* suggests a regulatory role in embryogenesis (Mercer, 1985). Ecdysteroids have also been found in cestodes like the sheep parasite *Moniezia expansa* and in the rat tapeworm *Hymenolepis diminuta* (Mercer et al., 1987b). Ecdysterone can be detected in the sera of infected hosts parasitized by *Schistosoma*, a trematode that synthesizes and releases the steroid to the host's circulation (Mercer, 1985). Although the role of ecdysteroids during insect metamorphosis had been widely demonstrated, the function of these steroids in parasites remains obscure.

### Sex steroids

Evidence for the steroidogenic capacity of *Schistosoma mansoni* was provided years ago by data showing the conversion of steroid precursors to its metabolites by the parasite homogenate (Briggs, 1972). We had shown that cultured *Trypanosoma cruzi* trypomastigotes synthesize androgens and estrogens from androstenedione and DHEA (Vacchina et al., 2008). We had also investigated the steroidogenic capacity of the cysticerci and tapeworm from *Taenia solium* and *Taenia crassiceps* WFU. The adult worm of *Taenia solium* and *crassiceps* WFU tapeworms are attached to the host gut with hooks that surrounds their head, and develop reproductive units called proglottids, where testis and ovaries gradually differentiate, and finally contain spermatocytes and infective eggs (Willms et al., 2003). *Taenia solium* cysticerci is the larval stage of the parasite and is found in the brain or muscle of humans and pigs, whereas *Taenia crassiceps* WFU cysticerci constitute a useful laboratory model due to their reproduction by budding in the peritoneal cavity of mice. We had been exploring the capacity of cysticerci to synthesize sex steroids *in vitro* and found that *T. solium* and *T. crassiceps* ORF cysticerci transform steroid precursors such as progesterone, dehydroepiandrostenedione, and androstenedione to androgens and estrogens (Gómez et al., 2000; Jiménez et al., 2006; Valdez et al., 2006). Other experiments showed that drugs that block steroidogenic enzymes interfered the *in vitro* steroid synthesis, suggesting the existence of this enzymes in the parasites (Aceves-Ramos et al., 2013).

Because of the presence of testis and ovaries in tapeworm proglottids, we had investigated the *in vitro* steroidogenic capacity of experimental *Taenia crassiceps* WFU and *solium* tapeworms obtained from the intestine of infected hamsters. *Taenia crassiceps* WFU tapeworms were incubated in the presence of ^3^H-DHEA and tritiated androstenediol and 17β-estradiol where recovered from the culture media, which strongly suggest the presence and activity of enzymes from the Δ5 steroid pathway in these tapeworms (Fernández Presas et al., 2008). *Taenia solium* tapeworms kept in culture also synthesized sex steroids (Valdez et al., 2014).

### Corticosteroids

Since the above mentioned literature indicated that *Taenia solium* and *Taenia crassiceps* cysticerci and tapeworms synthesized sex steroid hormones, we thought these organisms could also synthesize corticosteroids. Thereafter, we had incubated *Taenia crassiceps* cysticerci in the presence of ^3^H-progesterone and found an important transformation into deoxycorticosterone (Valdez et al., 2012), a steroid that has mineralocorticoid functions in vertebrates and also display some glucocorticoid properties. In addition, the parasites synthesized corticosterone, which was measured by radioimmunoassay in the culture media. More recently, we found corticosteroid-like synthesis in *Taenia solium* tapeworms (Valdez et al., 2014). It had been shown that in the adrenal reticular zone of mammals an excess of progesterone can be inactivated by 20HSD activity (Pelletier et al., 2005) and that cortisol and corticosterone can be inactivated by 11β-hydroxylase in the adrenal glomerulose zone (for a review see Odermatt and Atanasov, 2009) but to our knowledge these enzymes had not been investigated in *Taenia solium* or *crassiceps* WFU organisms. Besides their effects on the own parasite development and differentiation, the cysticerci, and tapeworm's steroidogenic capacity might play a role in the permanence of the parasites in muscle and brain tissues and in the host intestine.

## Parasites express steroidogenic enzymes

Some steroidogenic enzymes had been described in parasites. For instance, sterol-sterifying enzymes were found in *Toxoplasma gondee*, a protozoan incapable of cholesterol de novo synthesis (Lige et al., 2013). The presence of 3β-hydroxysteroid dehydrogenase in *Sarcocystis spp* had been shown by immunohistochemistry (Yarim et al., 2004); the enzyme is also present in *Taenia solium* cysticerci and tapeworms (Fernández Presas et al., 2008). Genes from the cholesterol synthesis pathway have been found and expressed in Giardia intestinalis (Hernandez and Wasserman, 2006). The parasite flatworm *Schistosoma japonicum* has a type 12 17-HSD that metabolizes estrone to estradiol (Zhou et al., 2009).

We have recently shown that *Taenia solium* cysticerci express the enzyme 17β-HSD that belongs to the short chain dehydrogenases/reductase family (Aceves-Ramos et al., 2014). Transient transfection of HEK293T cells with Tsol17β-HSD-pcDNA3.1 (+) induced expression of Tsol17β-HSD that transformed ^3^H-androstenedione into testosterone. In contrast, ^3^H-estrone was not significantly transformed into estradiol. Therefore, *Taenia solium* cysticerci express a 17β-HSD that catalyzes the androgen reduction and belongs to the short chain dehydrogenases/reductase (SDR) protein superfamily (Aceves-Ramos et al., 2014). Recently, a sequence (EmW000624600 which is available at: http://www.genedb.org/Homepage/Emultilocularis) with an identity of 84% with Tsol-17βHSD and a total coverage has been described for *E. multilocularis*, suggesting the presence of 17 HSD enzymes in this *Taeniid* family (Tsai et al., 2012). However, the expression level and enzyme activity has not been yet investigated.

## Concluding remarks

The knowledge of parasite endocrinology will contribute to our understanding of parasite biology and their interactions with the host. Sex steroids and corticosteroids are important hormones for the growth, differentiation, and performance in many species. Therefore, the synthesis of these hormones by parasites themselves may be critical for their own development and viability. In addition, it was shown that the production of steroids by parasites is regulated by corticosteroids and affected by steroidogenic inhibitors, which may be used as tools to combat the infection in some clinical situations. Furthermore, steroid synthesis by parasites may contribute to defend them from the attack of immune cells and therefore facilitate their survival in the host tissues.

### Conflict of interest statement

The authors declare that the research was conducted in the absence of any commercial or financial relationships that could be construed as a potential conflict of interest.

## References

[B1] Aceves-Ramos A. de la Torre P. Hinojosa L. Ponce A. García-Villegas R. Laclette J. P.. (2014). Cloning, characterization and functional expression of *Taenia solium* 17 beta-hydroxysteroid dehydrogenase. Gen. Comp. Endocrinol. 203, 186–192. 10.1016/j.ygcen.2014.03.021 24698785

[B2] Aceves-Ramos A. Valdez R. A. Gaona B. Willms K. Romano M. C. (2013). Steroid synthesis by *Taenia crassiceps* WFU cysticerci is regulated by enzyme inhibitors. Gen. Comp. Endocrinol. 188, 212–217. 10.1016/j.ygcen.2013.03.034 23608546

[B3] Al Qarawi A. A. (2005). Infertility in the dromedary bull: a review of causes, relations and implications. Anim. Reprod. Sci. 87, 73–92. 10.1016/j.anireprosci.2004.11.003 15885442

[B4] Bahr J. M. Frayha G. J. Hajjar J. J. (1979). Mechanism of cholesterol absorption by the hydatid cysts of *Echinococcus granulosus* (Cestoda). Comp. Biochem. Physiol. 62A, 485–490. 10.1016/0300-9629(79)90090-2

[B5] Bansal D. Bahatti H. S. Seghal R. (2005). Role of cholesterol in parasitic infections. Lipid. Health Dis. 9, 4–10. 10.1186/1476-511X-4-10 15882457 PMC1142336

[B6] Barthelemy M. Gabrion C. Petit G. (2004). Reduction in testosterone concentration and its effect on the reproductive output of chronic malaria-infected male mice. Parasitol. Res. 93, 475–481. 10.1007/s00436-004-1160-2 15243802

[B7] Bazin M. A. Loiseau P. M. Bories C. Letoumeux Y. Rault S. EI Kihen L. (2006). Synthesis of oxysterols and nitrogenous sterols with antileshmania and tripacidal activities. Eur. J. Med. Chem. 41, 1109–1116. 10.1016/j.ejmech.2006.03.033 16949702

[B9] Briggs M. H. (1972). Metabolism of steroid hormones by schistosomes. Biochim. Biophys. Acta 280, 480–485. 10.1016/0005-2760(72)90256-1 4643348

[B10] Carrero J. C. Cervantes C. Moreno-Mendoza N. Saavedra E. Morales-Montor J. (2006). Dehydroepiandrosterone decreases while cortisol increases *in vitro* growth and viability of *Entamoeba histolytica*. Microbes Infect. 8, 323–331. 10.1016/j.micinf.2005.06.030 16293437

[B11] Cleator M. Delves C. J. Howells R. E. Rees H. H. (1987). Identity and tissue localization of free and conjugated edcysteroids in adults of *Dirofilaria immitis* and *Ascaris surum*. Mol. Biochem. Parasitol. 25, 93–105. 10.1016/0166-6851(87)90022-3 3670345

[B12] Coutinho A. E. Chapman K. E. (2011). The anti-inflammatory and immunosuppressive effects of glucocorticoids, recent developments and mechanistic insights. Mol. Cell Endocrinol. 335, 2–13. 10.1016/j.mce.2010.04.005 20398732 PMC3047790

[B13] Dangel K. C. Keppel M. Tabujew K. Sures S. (2014). Effects of *Anguillicola novaezelandiae* on the levels of cortisol and hsp70 in the European Eel. Parasitol. Res. 113, 3817–3822. 10.1007/s00436-014-4049-8 25096532

[B14] de Loof A. Huybrechts R. (1998). “Insects do not have sex hormones”: a myth? Gen. Comp. Endocrinol. 111, 245–260. 10.1006/gcen.1998.7101 9707471

[B15] Dubansky B. Whitaker B. Galvez F. (2011). Influence of cortisol on the attachment and metamorphosis of larval *Utterbackia imbecillis* on Bluegill Sunfish (*Lepomis macrochirus*). Biol. Bull. 220, 97–106. 10.2307/23046932 21551446

[B16] Dunlap D. D. Schall J. J. (1995). Hormonal alterations and reproduction inhibition in male fence lizards (*Sceloporus occidentals*) infected with the malarial parasite *Plasmodium mexicanum*. Physiol. Zool. 68, 608–621.

[B16a] Escobedo G. Larralde C. Chavarria A. Cerbón M. A. Morales-Montor J. (2004). Molecular mechanisms involved in the differential effects of sex steroids on the reproduction and infectivity of *Taenia crassiceps*. J. Parasitol. 6, 1235–1244. 10.1645/GE-297R 15715212

[B17] Evershed R. P. Mercer J. G. Rees H. H. (1987). Capillary gas chromatography-mass spectrometry of ecdysteroids. J. Chromatogr. 390, 357–369. 10.1016/S0021-9673(01)94387-0 3584307

[B17a] Hernández-Bello R. Ramirez-Nieto R. Muñiz-Hernández S. Nava-Castro K. Pavón L. Sánchez-Acosta A. G.. (2011). Sex steroids effects on the molting process of the helminth human parasite *Trichinella spiralis*. J. Biomed. Biotechnol. 2011:625380. 10.1155/2011/625380 22162638 PMC3228608

[B20] Fernández Presas A. M. Willms K. Romano M. C. (2008). The key steroidogenic enzyme 3ß-hydroxysteroid dehydrogenase is present in the strobilae and larvae of *Taenia solium* and *T. crassiceps* WFU strain. Parasitol. Res. 103, 847–852. 10.1007/s00436-008-1066-5 18626663

[B21] Fleming M. W. (1985). *Ascaris suum;* role of ecdysteroids in molting. Exp. Parasitol 60, 207–210. 10.1016/0014-4894(85)90024-4 4029350

[B22] Foster J. M. Mercer J. G. Rees H. H. (1992). Analysis of ecdysteroids in the trematodes, *Schistosoma mansoni* and *Fasciola hepatica*. Trop. Med. Parasitol. 43, 239–244. 1293728

[B23] Gómez Y. Valdez R. A. Larralde C. Romano M. C. (2000). Sex steroids and parasitism. Taenia crassiceps cysticercus metabolizes exogenous androstenedione to testosterone *in vitro*. J. Steroid. Biochem. Mol. Biol. 74, 143–147. 10.1016/S0960-0760(00)00099-6 11086233

[B24] Hernandez P. C. Wasserman M. (2006). Do genes from the cholesterol pathway exist and express in *Giardia intestinalis*? Parsitol. Res. 98, 194–199. 10.1007/s00436-005-0039-1 16323024

[B25] Hinojosa L. Valdez R. A. Salvador V. Rodriguez A. G. Willms K. Romano M. C. (2011). The effect of corticosteroids on sex steroid synthesis in cultured *Taenia crassiceps* Wake Forest University (WFU) cysticerci. J. Helminthol. 86, 465–469. 10.1017/S0022149X11000708 22152276

[B26] Jiménez P. Valdéz R. A. Romano M. C. (2006). Metabolism of steroid hormones by *Taenia solium* and *Taenia crassiceps* cysticerci. J. Steroid Biochem. Mol. 99, 203–208. 10.1016/j.jsbmb.2006.01.002 16644209

[B27] Lafont R. (2000). The endocrinology of invertebrates. Ecotoxicology 9, 41–57 10.1023/A:1008912127592

[B28] Li M. Leatherland J. F. Woo P. T. K. (2013). Cortisol and dexamethasone increase the *in vitro* multiplication of the haemoflagellate *Cryptobia salmonistica*, possible by interaction with glucocorticoid receptor-like protein. Int. J. Parasitol. 43, 353–360. 10.1016/j.ijpara.2012.11.009 23262305

[B29] Li M. Patrick T. K. Woo P. T. K. (2014). Glucocorticoid receptors on and in a unicellular organism, *Cryptobia salmonistica*. Int. J. Parasitol. 44, 205–210. 10.1016/j.ijpara.2013.10.006 24333137

[B30] Lige B. Sampels V. Coppens I. (2013). Characterization of a second sterol-esterfying enzyme in Toxoplama highlights the importance of cholesterol storage pathways for the parasite. Mol. Microbiol. 87, 951–967. 10.1111/mmi.12142 23374239 PMC3581733

[B31] Lim A. Kumar V. Hari Dass S. A. Vyas A. (2013). *Toxoplasma gondee* infection enhances testicular steroidogenesis in rats. Mol. Ecol. 22, 102–110. 10.1111/mec.12042 23190313

[B32] Lingnau A. Margos G. Maier W. A. Seitz H. M. (1993). The effects of hormones on the gametocytogenesis of *Plasmodium falciparum in vitro*. Appl. Parasitol. 34, 153–160. 8220571

[B33] Madison B. N. Woo P. T. Bernier N. J. (2013). Duress without stress: cryptobia infection results in HPI disfunction in rainbow trout. J. Endocrinol. 218, 287–297. 10.1530/JOE-13-0155 23814015

[B18] Magaraci F. Jiménez C. J. Rodrigues C. Rodrígues J. C. Braga M. V. Yardley V.. (2003). Azasterols as inhibitors of sterol 24-methyltransferase in *Leishmania* species and *Tryposoma cruzi*. J. Med. Chem. 46, 4714–4727. 10.1021/jm021114j 14561091

[B35] Mendis A. H. Rees H. H. Gooswin T. W. (1984). The occurrence of ecdysteroid in the cestode. Monieza expansa. Mol. Biochem. Parasitol. 10, 123–138. 10.1016/0166-6851(84)90001-X 6700637

[B36] Mendis A. H. Rose M. E. Rees H. H. Goodwin T. W. (1983). Ecdysteroids in adults of the nematode, *Dirofilaria immitis*. Mol. Biochem. Parasitol. 9, 209–226. 10.1016/0166-6851(83)90098-1 6674810

[B37a] Mercer J. G. (1985). Developmental hormones in parasitic helminths. Parasitol. Today 1, 96–100. 15275591 10.1016/0169-4758(85)90002-x

[B37] Mercer J. G. Barker G. C. Howells R. E. Rees H. H. (1990). Investigation of ecdysteroid excretion by adult *Dirofilaria immitis* and *Brugia pahangi*. Mol. Biochem. Parasitol. 38, 89–95. 10.1016/0166-6851(90)90208-4 2320053

[B38] Mercer J. G. Munn A. E. Rees H. H. (1987a). *Echinococcus granulosus*: occurrence of ecdcysteroids in protoscoleces and hydatid cyst fluid. Mol. Biochem. Parasitol. 24, 203–214. 10.1016/0166-6851(87)90107-1 3627169

[B39] Mercer J. G. Munn A. E. Rees H. H. (1987b). Analysis of ecdsysteroids in different developmental stages of *Hymenolepis diminuta*. Mol. Biochem. Parasitol. 25, 61–71. 10.1016/0166-6851(87)90019-3 3670343

[B40] Morales J. Larralde C. Arteaga M. Govezensky T. Romano M. C. Moralí G. (1996). Inhibition of sexual behavior in male mice infected with *Taenia Crassiceps* cysticerci. J. Parasitol. 82, 689–693. 10.2307/3283875 8885872

[B41] Morales-Montor J. Chavarria A. de Leon M. A. del Castillo L. I. Escobedo E. G. Sanchez E. N.. (2004). Host gender in parasitic infections of mammals: an evaluation of the female host supremacy paradigm. J. Parasitol. 90, 531–546. 10.1645/GE-113R3 15270097

[B42] Morales-Montor J. Hallal-Caballeros C. Romano M. C. Damian R. T. (2002). Inhibition of P450-aromatase prevents feminization and induces protection during cisticercosis. Int. J. Parasitol. 32, 1379–1387. 10.1016/S0020-7519(02)00130-3 12350373

[B43] Morrison D. D. Vandee Waa E. Bennett J. L. (1986). Effects of steroids and steroid synthesis inhibitors on fecundity of *Schistosoma mansoni in vitro*. J. Chem. Ecol. 12, 1901–1908. 10.1007/BF01022391 24305903

[B44] Odermatt A. Atanasov A. G. (2009). Mineralocorticoid receptors: emerging complexity and functional diversity. Steroids 74, 163–171. 10.1016/j.steroids.2008.10.010 19022273

[B45] Pelletier G. Luu-The V. Li S. Labrie F. (2005). Localization of type 5 17*β*-hydroxysteroid dehydrogenase mRNA in mouse tissues as studied by *in situ* hybridization. Cell Tissue Res. 320, 393–398. 10.1007/s00441-005-1105-9 15846505

[B46] Popiel I. Basch P. F. (1986). *Schistosoma mansoni* colesterol uptake by paired and unpaired worms. Exp. Parasitol. 61, 343–347. 10.1016/0014-4894(86)90189-X 3709750

[B47] Reyes-Hernandez J. L. Leung G. McKay D. M. (2013). Cestode regulation of inflammation and inflammatory research. Int. J. Parasitol. 43, 233–243. 10.1016/j.ijpara.2012.09.005 23058631

[B48] Roberts C. W. Walker W. Alexander J. (2001). Sex-associated hormones and immunity of protozoan parasites. Clin. Microbiol. Rev. 14, 476–488. 10.1128/CMR.14.3.476-488.2001 11432809 PMC88985

[B49] Romano M. C. Valdez R. A. Cartas A. L. Gómez Y. Larralde C. (2003). Steroid hormone production by parasites: the case of *Taenia crassiceps* and *Taenia solium* cysticerci. J. Steroid Biochem. Mol. Biol. 85, 221–225. 10.1016/S0960-0760(03)00233-4 12943707

[B49a] Rumjanek F. D. Simpson A. J. G. (1980). The incorporation and utilization of radiolabelled lipids by adult *Schistosoma mansoni in vitro*. Mol. Biochem. Parasitol. 1, 31–44. 7442709 10.1016/0166-6851(80)90039-0

[B14a] Santos C. D. Toldo M. P. Levy A. M. Prado J. C. Jr. (2008). *Trypanosoma cruzi*: effects of social stress in *Calomys callosus* a natural reservoir of infection. Exp. Parasitol. 119, 197–201. 10.1016/j.exppara.2008.01.011 18387609

[B50] Silveria A. M. Friche A. A. Rumjanek F. D. (1986). Transfer of (14C) cholesterol and its metabolities between adult male and female worms of *schistosoma mansoni*. Comp. Biochem. Physiol. B 85, 851–857. 10.1016/0305-0491(86)90186-0 3816158

[B51] Simons K. Toomre D. (2000). Lipids rafts signal transduction. Nat. Rev. 1, 31–39. 10.1038/35036052 11413487

[B53] Thompson S. N. Kavaliers M. (1994). Physiological basis for parasite induced alterations of host behavior. Parasitology 109, S119–S138. 10.1017/S0031182000085139 7854846

[B54] Tsai I. J. Zarowiecki M. Holroyd N. Garciarrubio A. Sanchez-Flores A. Brooks K. L.. (2012). The genomes of four tapeworm species reveal adaptations to parasitism. Nature 496, 57–63. 10.1038/nature12031 23485966 PMC3964345

[B55] Urbina J. A. Conception J. L. Rangel S. Visval G. Lira R. (2002). Mol. Squalene synthase as a chemotherapeutic target in *Tripsosoma cruzi* and *Leishmania mexicana*. Biochem. Parasitol. 125, 35–45. 10.1016/S0166-6851(02)00206-2 12467972

[B56] Vacchina P. Valdez R. A. Revelli S. Romano M. C. (2008). Steroidogenic capacity of *Trypanosome cruzi* trypomastigotes. J. Steroid Biochem. Mol. Biol. 111, 282–286. 10.1016/j.jsbmb.2008.06.016 18640275

[B57] Valdez R. A. Hinojosa L. Gómez Y. Willms K. Romano M. C. (2012). *Taenia crassiceps* WFU cysticerci synthesize corticosteroids *in vitro*: metyrapone regulates the production. Comp. Endocrinol. 176, 409–414. 10.1016/j.ygcen.2012.01.015 22321721

[B58] Valdez R. A. Jiménez P. Cartas A. L. Gómez Y. Romano M. C. (2006). *Taenia solium* cysticerci synthesize androgens and estrogens *in vitro*. Parasitol. Res. 98, 472–476. 10.1007/s00436-005-0095-6 16416116

[B59] Valdez R. A. Jiménez P. Fernández-Presas A. M. Aguilar L. Willms K. Romano M. C. (2014). *Taenia solium* tapeworms synthesize corticosteroids and sex steroids *in vitro*. Gen. Comp. Endocrinol. 205, 62–67. 10.1016/j.ygcen.2014.04.014 24793221

[B60] Wang T. Gao J.-M. Yi S. Q. Geng G. Q. Gao X. J. Shen J. L.. (2014). The growth rate of *Toxoplasma gondii*, a protozoan parasite, increased in the peritoneal macrophages of rats treated with glucocorticoids *in vivo*. Parasitol. Res. 113, 351–358. 10.1007/s00436-013-3661-3 24248630

[B61] Willms K. Caro J. A. Robert L. (2003). Ultrastructure of spermatogonia and spermatocyte lobules in *Taenia solium* strobilac (Cestoda, *Cyclophyllidea, Taeniidae*) from golden hamsters. Parasitol. Res. 90, 479–488. 10.1007/s00436-003-0897-3 12827507

[B62] Yarim M. Yildiz K. Kabakci N. (2004). Immunohistochemical localization of 3β-hydroxysteroid dehydrogenase in Sarcocystis sp. Parasitol. Res. 93, 457–460. 10.1007/s00436-004-1152-2 15243798

[B63] Zhou Y. Zheng H. Chen Y. Zhang L. Wang K. Guo J. Huang Z. (2009). The *Schistosoma japonicum* genome reveals features of host-parasite interplay. Nature 460, 345–351. 10.1038/nature08140 19606140 PMC3747554

